# Late stage definitive endodermal differentiation can be defined by Daf1 expression

**DOI:** 10.1186/s12861-016-0120-2

**Published:** 2016-05-31

**Authors:** Soichiro Ogaki, Hisayoshi Omori, Mayu Morooka, Nobuaki Shiraki, Seiichi Ishida, Shoen Kume

**Affiliations:** School of Life Science and Technology, Tokyo Institute of Technology, 4259-B-25 Nagatsuta-cho, Midori-ku, Yokohama, Kanagawa 226-8501 Japan; Stem Cell Biology, Institute of Molecular Embryology and Genetics, Kumamoto University, Honjo 2-2-1, Kumamoto, 860-0811 Japan; Division of Pharmacology, National Institute of Health Science, 1-18-1 Kamiyoga Setagaya-ku, Tokyo, 158-8501 Japan

**Keywords:** Pluripotent stem cell, Definitive endoderm, Daf1, In vitro differentiation, Proliferation, Adhesion

## Abstract

**Background:**

Definitive endoderm (DE) gives rise to the respiratory apparatus and digestive tract. Sox17 and Cxcr4 are useful markers of the DE. Previously, we identified a novel DE marker, Decay accelerating factor 1(Daf1/CD55), by identifying DE specific genes from the expression profile of DE derived from mouse embryonic stem cells (ESCs) by microarray analysis, and in situ hybridization of early embryos. Daf1 is expressed in a subpopulation of E-cadherin + Cxcr4+ DE cells. The characteristics of the Daf1-expressing cells during DE differentiation has not been examined.

**Results:**

In this report, we utilized the ESC differentiation system to examine the characteristics of Daf1-expressing DE cells. We found that Daf1 expression could discriminate late DE from early DE. Early DE cells are Daf1-negative (DE-) and late DE cells are Daf1-positive (DE+). We also found that Daf1+ late DE cells show low proliferative and low cell matrix adhesive characteristics. Furthermore, the purified SOX17^low^ early DE cells gave rise to Daf1+ Sox17^high^ late DE cells.

**Conclusion:**

Daf1-expressing late definitive endoderm proliferates slowly and show low adhesive capacity.

**Electronic supplementary material:**

The online version of this article (doi:10.1186/s12861-016-0120-2) contains supplementary material, which is available to authorized users.

## Background

The definitive endoderm (DE) gives rise to the gastrointestinal and digestive system. In the mouse embryo, the DE progenitors reside at the posterior region of the epiblast that derived from the inner cell mass [1]. During gastrulation, as the cells ingress through the primitive streak, the epiblast segregates into the three germ layers that form the somatic cell lineages of the ectoderm, mesoderm, and definitive endoderm (DE). DE arises from the *Forkhead box A2 (Foxa2*)-expressing anterior primitive streak (APS) [2–5] and is then regionalized into the fore-, mid-, and hindgut [6].

The DE is identified by the expression of *SRY (sex-determining region Y)-box 17* (*Sox17*) [7–9] and chemokine (C-X-C motif) receptor 4 (Cxcr4) [10–12]. *Sox17* mutant mouse embryos have a reduced DE, apoptosis of the foregut, and abnormal morphogenesis of the mid- and hindgut [9]. Sox17 is also required for the assembly of the basement membrane, as the *Sox17* mutant embryo fails to segregate the DE from the mesoderm [13]. Activin is a frequently used inducer for DE differentiation from pluripotent stem cells, embryonic stem cells (ESCs), and induced pluripotent stem cells (iPSCs) [14–16]. When SOX17 is overexpressed, human ESCs spontaneously differentiate into the DE, independent of Activin [17]. In zebrafish embryos, *cxcr4a* regulates directional migration [11, 18] and DE proliferation during gastrulation [19]. In chick embryos, *Cxcr4* is expressed in the DE and angioblasts. Cxcr4 and its ligand Cxcl12 form a reciprocal signaling loop that triggers angioblast migration to the pancreatic endoderm and induces pancreatic development. Inhibition of Cxcr4 suppresses angioblast migration into the pancreatic endoderm region. As a result, the size of the pancreas decreases [10]. Although Cxcr4 is also expressed in the mesodermal cells, it is often used in combination with E-cadherin for purifying ESC-derived DE cells using flow cytometry [12].

Daf1 is an inhibitor of complementary activation [20]. Daf1 is expressed by immune cells and DE-derived tissue, such as intestine and airway [21]. Using microarray analysis and in situ hybridization, we previously identified Daf1 as a DE cell surface marker based on its expression in ESC-derived and embryonic DE. Daf1 is also expressed in pancreatic progenitor cells [22, 23]. However, the role of Daf1 in the DE is not well understood. In this study, we found that the DE population that expresses Daf1 (Daf1 + DE) has slow cell cycling and low cell-matrix adhesive characteristics. Furthermore, Daf1-negative DE cells (Daf1-DE) turn out to be Foxa2 + Sox17^low^ cells and Daf1-positive DE (Daf1 + DE) cells to be Foxa2 + SOX17^high^ cells. Our results therefore suggest that E-cadherin + Cxcr4 + DE is composed of two populations: Sox17^low^ early DE and Sox17^high^ late DE. Sox17^high^ late DE cells were positive for Daf1, and were slow proliferative and low cell-matrix adhesive cells.

## Results

### Daf1 + DE are slowly proliferating cells

**Fig. 1:**
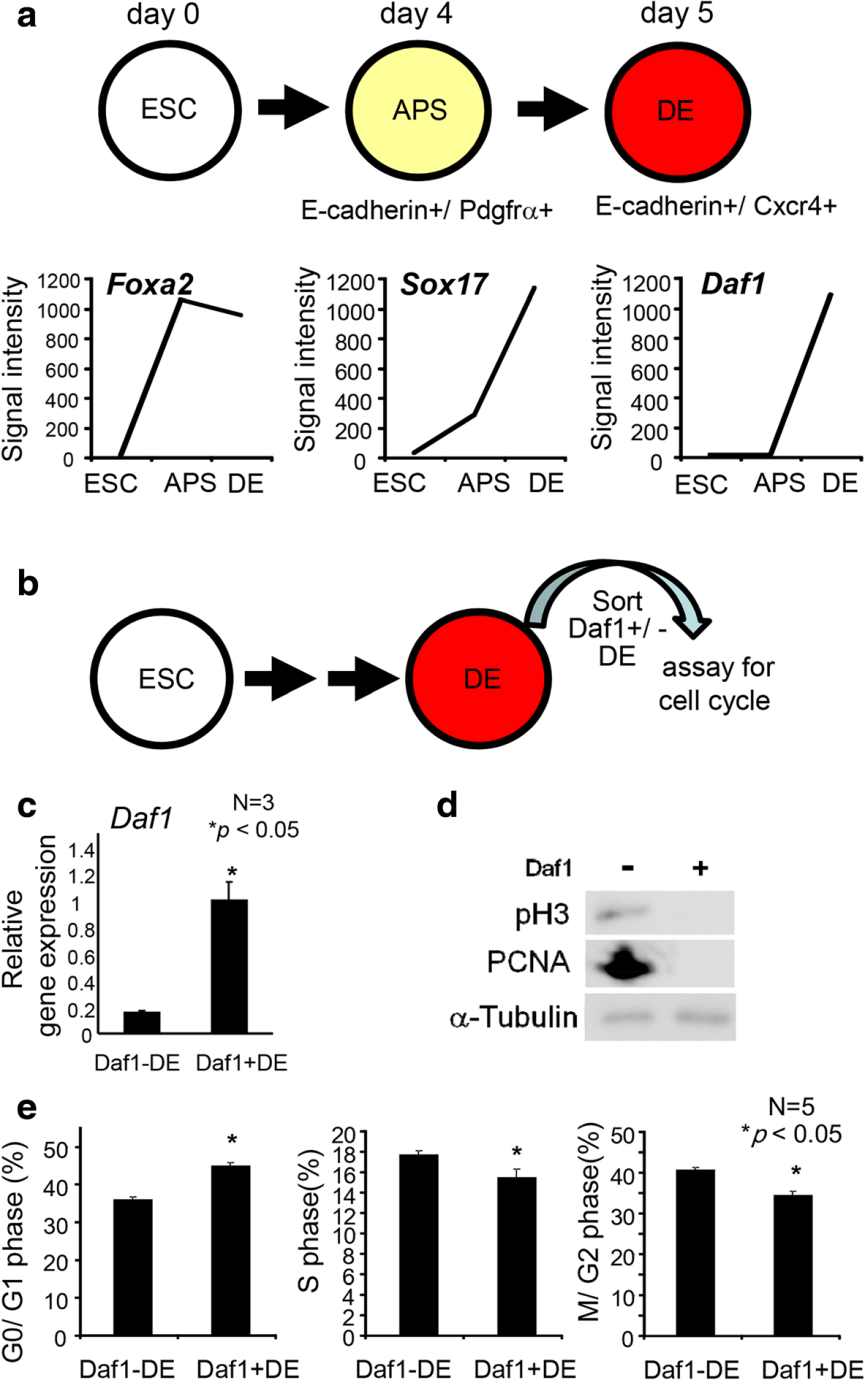
Daf1+ DE cells are less proliferative than Daf1- DE cells. **a** Mouse ESCs (ESC) were differentiated first to the anterior primitive streak then into the definitive endoderm (DE). Our previously reported microarray analysis of *Foxa2*, *Sox17* and *Daf1* expression during DE differentiation are plotted in a time dependent manner. Daf1 expression increased in the DE but not in the APS. ESC; mouse embryonic stem cells, APS; anterior primitive streak, DE; definitive endoderm. **b** Schematic drawing of the experiment to analyze cell cycle phases of the sorted DE. ES cells were differentiated into DE, then dissociated and sorted for Daf1+/- cells. The sorted cells were immediately analyzed for cell cycle. **c** Real time-PCR analysis showed that *Daf1* expression was enriched in Daf1+ DE. Y axis shows relative gene expression, with 1 equivalent to *Daf1* expression level in Daf1 + DE cells. **d**, **e** Proliferative properties of Daf1 + DE and Daf1-DE cells were assayed. **d** Cell proliferation marker, pH3 (M phase), and PCNA (every cell phases except G0) was down-regulated in Daf1 + DE. Analyzed by western blot analysis. **e** Cell cycle analysis revealed that Daf1 + DE reside longer in the G0/G1 phase with a shorter S and M/G2 phases compared to that of the Daf1-DE cells. Raw data are shown in Additional file 2. Student’s two tailed *t*-test

Previously, we reported Daf1 as a surface marker, expressed in a subpopulation of DE [23]. DE are defined as E-cadherin+/Cxcr4+ cells [12]. When cultured in Activin-containing medium [24, 25], ESCs sequentially give rise to APS cells on day4 (defined as E-cadherin+/Pdgfra + cells), then to DE cells (defined as E-cadherin+/Cxcr4+ cells) on day 5 (Fig. 1). A plot of our previous microarray analysis results of the APS and DE cells [23, 24] shows the time dependent expression of Foxa2, Sox17 and Daf1 (Fig. 1a). *Foxa2* was highly expressed in the APS and DE. *Sox17* was lowly expressed in the APS and highly expressed in the DE. *Daf1* expression was absent in the APS and present in the DE (Fig. 1a). We then analyzed Daf1+ cells for the expression of *Nanog*, a pluripotent marker, using a mouse Nanog-iPS cell line bearing a *green fluorescent protein* (GFP) reporter driven under *Nanog* promoter. Daf1-positive cells turned out to be *Nanog*/GFP negative (Additional file 1). Taken together, Daf1 marks a subpopulation of DE and is first expressed in the DE, but not earlier in the APS.

To identify the differences between Daf1 + DE and Daf1-DE cells, we purified Daf1+/-DE cells and compared their properties. Real time PCR analysis of the sorted Daf1+/-DE cells confirmed *Daf1* expression is enriched in Daf1 + DE (Fig. 1b, c). Expressions of an M phase marker, phosphorylated histone H3 (pH3) or Proliferating Cell Nuclear Antigen (PCNA) that marks proliferating cells at every phase of the cell except G0 were enriched in Daf1-DE than in Daf1 + DE cells, revealed by western blot analysis (Fig. 1d). We analyzed the cell cycle phases and found that Daf1 + DE cells showed a longer Go/G1 phase and shorter S, M/G2 phase compared to Daf1-DE cells (Fig. 1e, Additional file 2). This suggested that cell proliferation was decreased in Daf1 + DE.

### Daf1 + DE are low adhesive cells

**Fig. 2:**
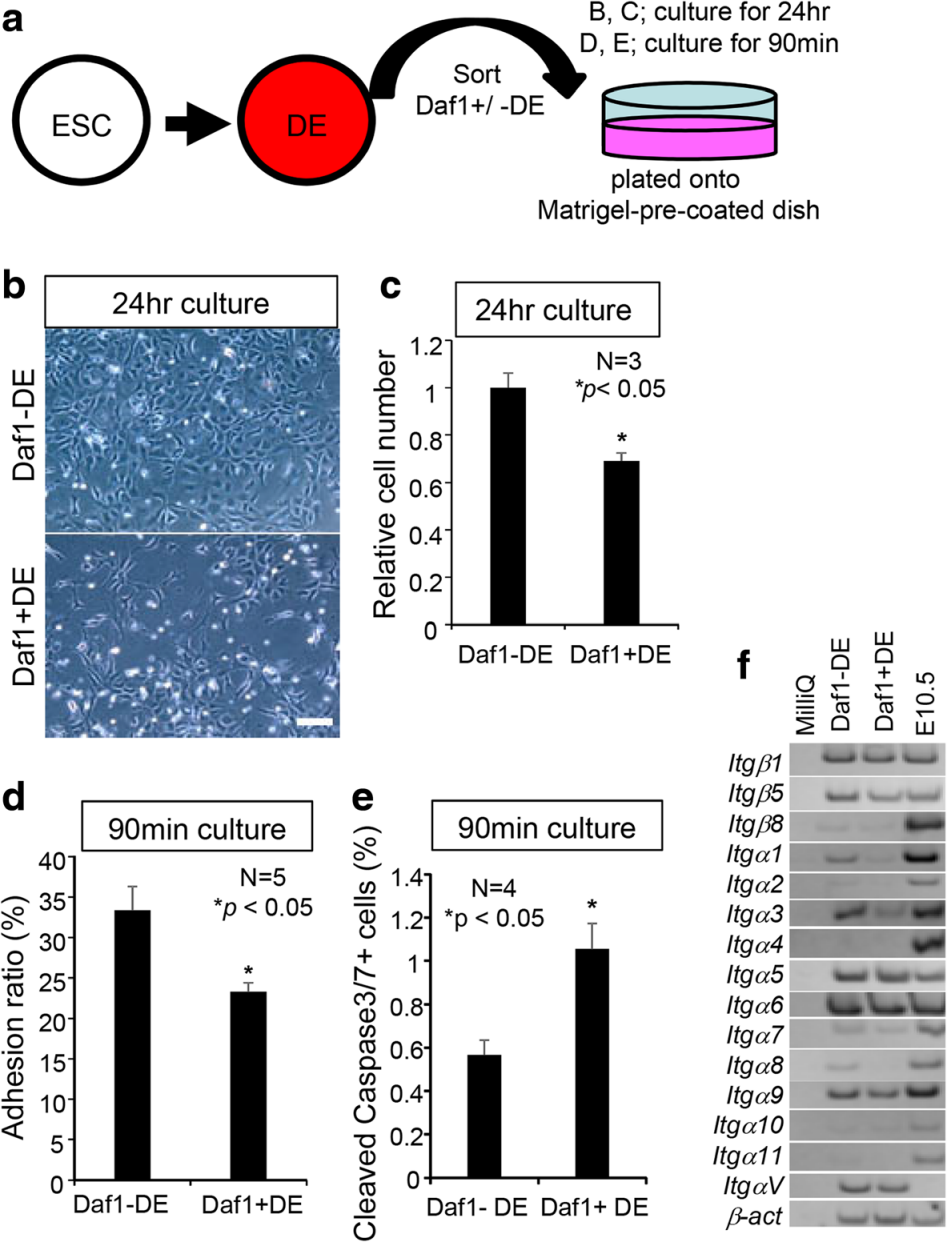
Daf1+ DE cells have lower cell-matrix adhesion than Daf1- DE cells. **a** A schematic drawing showing experimental design. The sorted DE cells were plated onto matrigel-pre-coated dishes and cultured for 24 h (**b**, **c**) or 90 min (**d**, e). **b** Transmission images of the cells derived from the Daf1+/-DE cells after cultured for 24 h. **c** After 24 h culture, the Daf1 + DE cells gave rise to fewer cells. **d** Cell-matrix adhesion assay, after 90 min culture. Quantification of the attached cells. The adhesion ability of Daf1 + DE cells onto matrigel is weaker than that of the Daf1-DE cells. **e** Quantification of Cleaved caspase3/7+ apoptotic cells. The Daf1 + DE cells gave rise to a larger proportion of apoptotic cells. **f** The *Integrin* expression profiles of the DE cells detected by RT-PCR analysis. Student’s two tailed *t*-test. Scale bars; 100 μm

Next, we performed flow cytometry using antibody against Daf1and plated at a same cell number of the sorted DE cells (Daf1-DE or Daf1 + DE) onto matrigel-pre-coated dishes (Fig. 2a). After 24 h culture, the Daf1+ DE gave rise to fewer cell number compared to that of Daf1-DE (Fig. 2b, c). The results suggested a higher level of cell death and/or lower plating efficiency of Daf1 + DE cells. We then examined cell-matrix adhesion and the extent of apoptosis, in which cells were allowed to adhere to the plate for 90 mins [26, 27] [28]. Our results revealed that Daf1 + DE cells showed a lower matrix adhesion ratio than Daf1-DE (Fig. 2d), and that Daf1 + DE cells were more apoptotic and showed a higher Cleaved Caspase3/7+ ratio (Fig. 2e) than Daf1-DE. Cells bind to extracellular matrix (ECM) through the ECM receptor, integrin [29, 30]. We found that expression of several subtypes of the *Integrin* genes, such as *Itgα1*, *Itgα3*, and *Itgα8,* was down-regulated in Daf1 + DE cells (Fig. 2f). Our results suggest that Daf1 + DE cells have lower adhesion capacity than Daf1-DE cells, which led to a lower plating efficiency and triggered cell death in the attached cells, compared to Daf1-DE cells.

### Daf1 is a marker of late stage DE

**Fig. 3:**
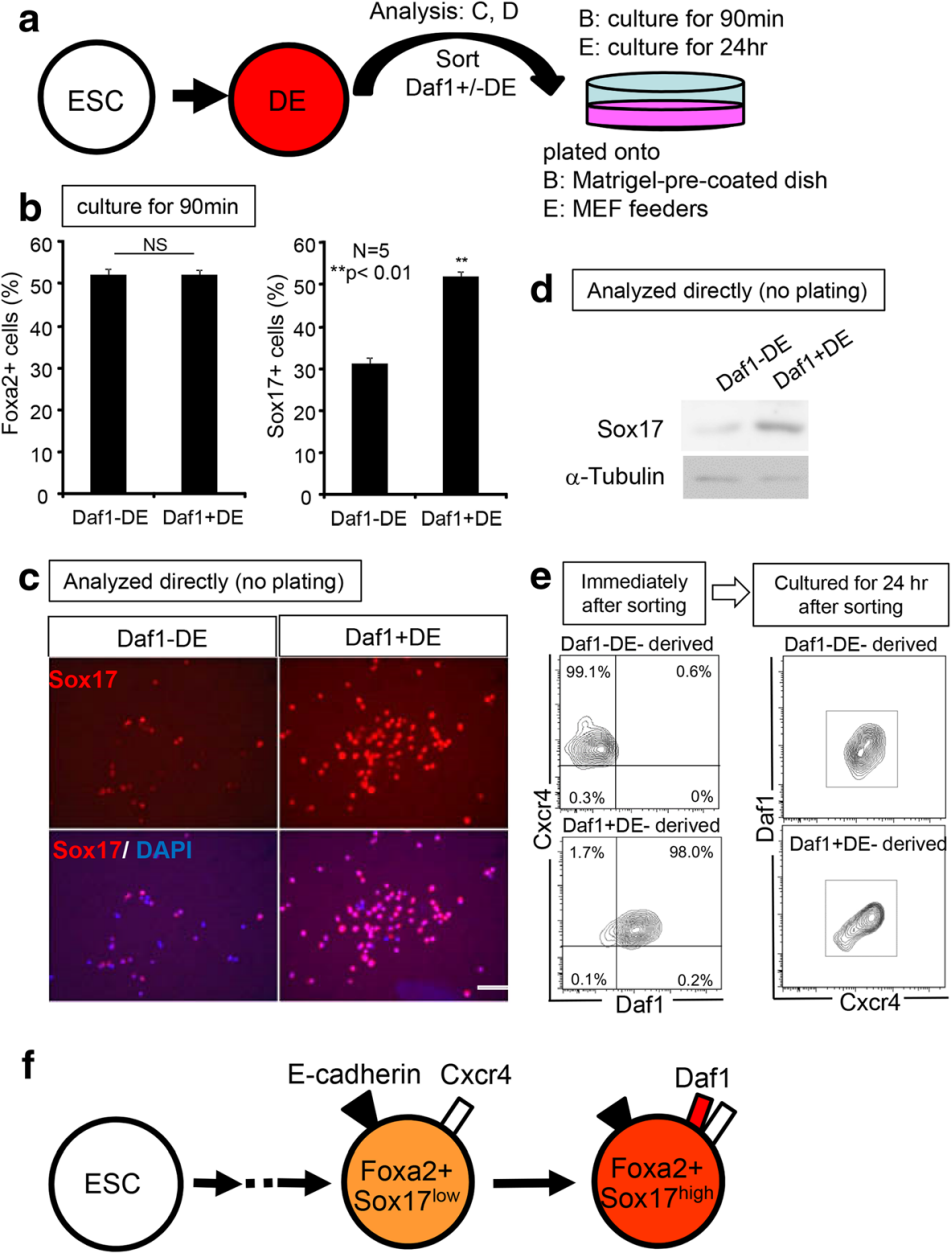
Daf1-DE is the progenitor of Daf1+ DE. **a** A schematic drawing showing experimental design. **b** Quantifications of Foxa2+ and Sox17+ cells by immunocytochemistry after 90 min cell-matrix adhesion assay. **c**, **d** The proportion of cells expressing Sox17 was higher in the Daf1 + DE as detected by immunocytochemical analysis (**c**), and the level of Sox17 expression was higher in the Daf1 + DE as detected by western blot (**d**), of the sorted DE cells (without plating). **e** Flow cytometry analysis of the descendent cells of Daf1-DE and Daf1 + DE. Left panels: Daf1, Cxcr4 expression profiles of the sorted Daf1+/- DE cells. Right panels: both Daf1-DE and Daf1 + DE acquired Daf1 expression after 24 h. **f** The scheme of Fig. 3. Sox17^low^ Daf1-DE differentiated into Sox17^high^ Daf1 + DE. Student’s two tailed *t*-test. Scale bar; 100 μm

To characterize the adherent cells onto Matrigel- pre-coated dish, we assayed for the expression of the DE markers, Foxa2 and Sox17, immediately after the adhesion assay (Fig. 3). Interestingly, whereas both Daf1+/-DE populations showed similar capacity for giving rise to Foxa2-expressing cells, Daf1 + DE cells showed a higher capacity for giving rise to Sox17-expressing cells (Fig. 3a, b). Foxa2 is expressed in both the APS and DE, whereas Sox17 is expressed at a higher level in the DE. We then confirmed Sox17 protein expression of the sorted Daf1+/-DE cells directly (without plating) by immunocytochemical analysis (Fig. 3c). Sox17 expression was found in a higher proportion and seemingly higher level in the sorted Daf1 + DE cells compared to Daf1-DE cells (Fig. 3c). Western blot analysis confirmed a higher level of Sox17 expression in the Daf1 + DE cells (Fig. 3d). Since Foxa2 expression is detected prior to that of Sox17, we then asked if Daf1-DE cells represent early DE and Daf1 + DE cells represent late DE, by examining if Daf1-DE cells differentiate into Daf1 + DE. Daf1+/-DE cells sorted by flow cytometry (purity >97.8 %, Daf1 and Cxcr4 expression profiles shown in Fig. 3e) were re-cultured on mouse embryonic fibroblast (MEF) feeders with Activin containing endoderm differentiation medium. We examined the expression of Daf1. To exclude MEF feeders, we analyzed E-cadherin + cells. Almost all of the Daf1-DE cells acquired Daf1 expression over the course of 24 h and gave rise to Daf1 + DE (>96.7 %), whereas Daf1 + DE cells did not turn into Daf1-DE (Fig. 3e). Taken together, these results suggest that Daf1-DE represent early and Daf1 + DE represent late DE cells during DE differentiation and that Daf1-DE become Daf1 + DE cells (Fig. 3f).

### Daf1+/-DE cells can give rise to pancreatic and intestinal fates

**Fig. 4:**
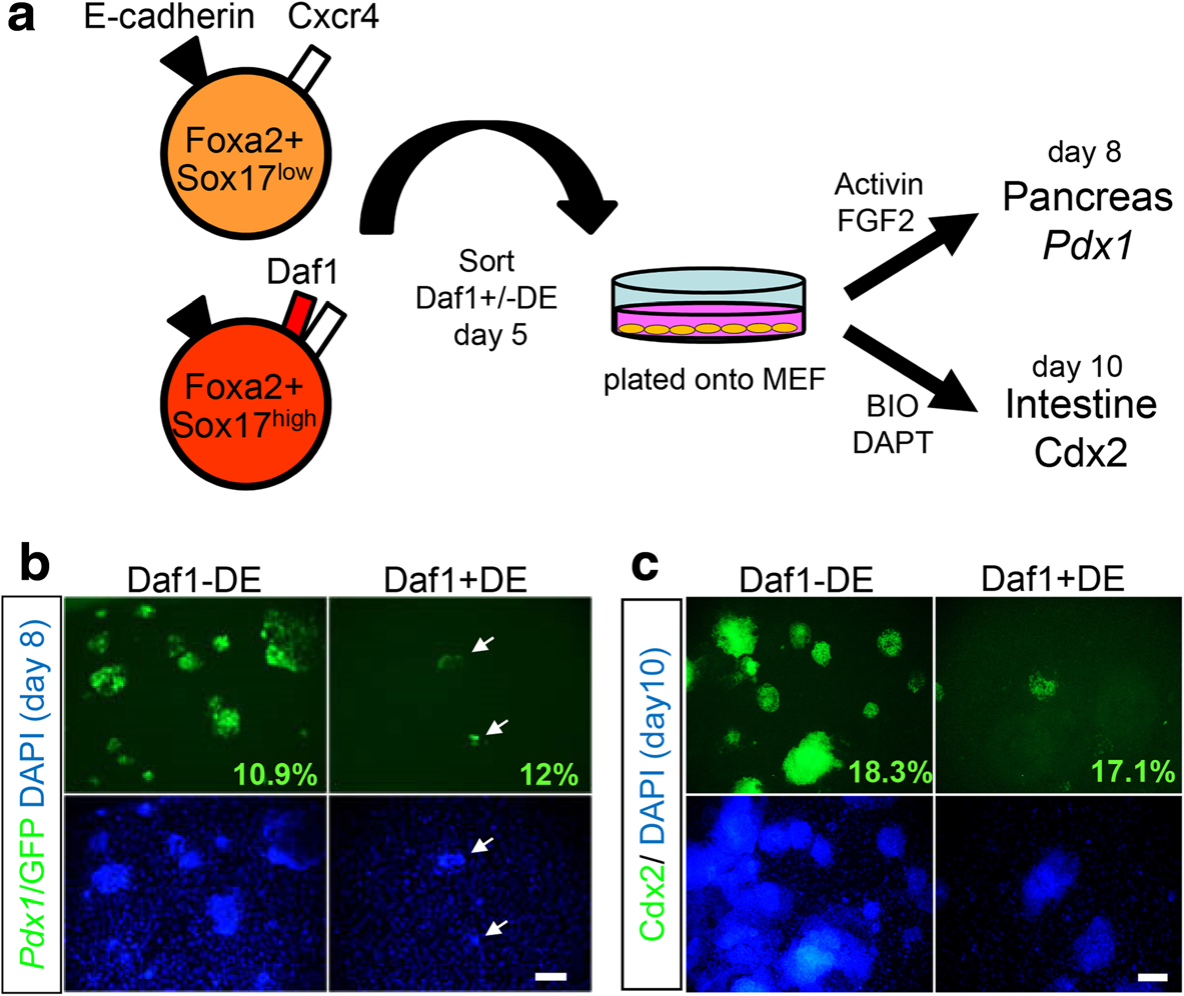
Both Daf1+/- could differentiate into pancreatic and intestinal fates. **a** A schematic drawing showing experimental design. DE was sorted on day 5. The sorted cells were plated onto MEF feeders and cultured with pancreatic and intestinal differentiation medium. Daf1- or Daf1 + DE cells differentiated into *Pdx1*/GFP+ cells after 4 days culture (**b**) and Cdx2+ cells after 5 days culture (**c**). **b** Arrows depict small colonies of Daf1 + DE-derived *Pdx1*/GFP+ cells. Numbers depict differentiation ratios: Daf1-DE-derived *Pdx1*/GFP+ cells; 10.9 %, Daf1 + DE-derived *Pdx1*/GFP+ cells; 12 % (day 9). **c** Numbers depict differentiation ratios: Daf1-DE-derived Cdx2+ cells; 18.3 %, Daf1 + DE-derived Cdx2+ cells; 17.1 % (day 10). Scale bar; 100 μm

We identified that Daf1-DE cells acquired Daf1 expression and turned into Daf1 + DE. If Daf1-DE represents early DE and Daf1 + DE represents late DE, both DE cells could give rise to regional endoderm derivatives of the gut. We next examined the abilities of the Daf1+/-DE cells to differentiate into pancreatic or intestinal cells. To visualize pancreatic differentiation, we used *Pdx1*/GFP mouse ESCs, in which GFP expression is driven by the *Pdx1* promoter [25]. We isolated Daf1+/-DE cells by flow cytometry (purity > 98 %), seeded the cells on MEF feeders, and cultured in the presence of Activin and FGF2 (Fig. 4a) [25]. We found that both Daf1- DE and Daf1+ cells gave rise to *Pdx1*/GFP+ cells, after plated for 3 days (day 8). The Daf1 + DE-derived cells yielded less *Pdx1*/GFP cells compared to that of the Daf1-DE cells (Fig. 4b, c). This discrepancy was probably due to a lower proliferation of the Daf1 + DE cells compared to Daf1-DE cells. Since the pancreas derives from the anterior DE, we then asked if the Daf1+/-DE cells could also differentiate into the posterior DE derivative of the intestine. DE derived from R1 ESCs were sorted and seeded onto MEF feeders and cultured at the presence of BIO and DAPT [16, 31]. After five days, both Daf1-DE and Daf1 + DE cells differentiated into Cdx2+ intestinal cells. Similar to pancreatic differentiation, Daf1 + DE cells are less proliferative and gave rise to small Cdx2+ colonies than Daf1-DE cells (Fig. 4c). Our results therefore suggest that both Daf1+/- DEs are capable of differentiating into the endodermal derivatives of the pancreas and intestine.

**Fig. 5:**
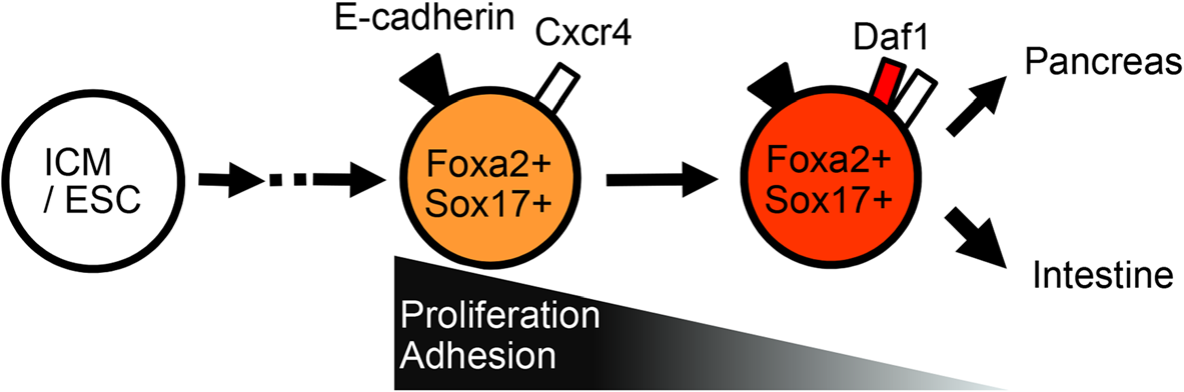
Proposed role of Daf1 in DE differentiation. ICM/ESC differentiated into the E-cadherin + Cxcr4 + Foxa2 + Sox17^low^Daf1- early DE. These cells then differentiated into the E-cadherin + Cxcr4 + Foxa2 + SOX17^high^Daf1+ late DE. The late DE differentiated into regionalized anterior (pancreatic) and posterior (intestinal) endodermal lineages. Transition from the Daf1-DE to Daf1 + DE is accompanied by restricted cell proliferation and cell-matrix adhesion. ICM; inner cell mass

## Discussion

Previously, we identified that Daf1 is expressed in the DE. Here, we identified Daf1 as a late DE marker. Daf1 is an inhibitor of complement activation [20]. Daf1 is expressed in the kidney, spleen, testis, intestine, and bronchi of the adult mouse [21]. *Daf1* deficiency is reported in autoimmune hemolytic anemia patients [32]. In *Daf1* knockout mice, IFN-γ expression increases, resulting in enhanced T cell response autoimmunity [33]. However, gastrointestinal-tract develops normally in *Daf1* knockout mice. Here, we examined the detailed expression patterns during DE differentiation using ESCs.

DE cells are defined as E-cadherin+/Cxcr4+ cells [12]. However, both E-cadherin and Cxcr4 are also expressed in the primitive streak [34, 35]. Therefore, the use of E-cadherin+/Cxcr4+ as a marker to define the DE cells is confined to a limited time window. Moreover, E-cadherin+/Cxcr4+ DE cells are a heterogeneous population. Here we used Daf1 to characterize a subpopulation of E-cadherin+/Cxcr4+ DE cells. We revealed that both Daf1-DE cells represent early DE and Daf1 + DE represent late DE. Daf1-DE and Daf1 + DE cells can give rise to the pancreatic and intestinal lineages. Daf1 + DE formed small colonies, due to their less proliferative and low adhesive characteristics than that of Daf1-DE cells (Fig. 5). A slight decrease in S, M/G2 phase and increase in G0/G1 phase in Daf1 + DE cells might reflect their property as more differentiated cells. Daf1 + DE cells seem to differentiate as efficiently into Pdx1-expressing cells, but not as efficiently into Cdx2-expressing cells, compared to Daf1-DE cells. This might due to a partial loss in differentiation potency of Daf1+ DE cells into the intestinal fate. We previously reported that regional-specific endodermal fates are determined sequentially in the order of stomach, intestine and pancreas, in the chick embryos [36]. It is possible that Daf1 + DE gradually lose potency to differentiate into intestinal lineages, but retains differentiation potency into pancreatic lineages, compared to that of Daf1-DE.

Cell-matrix adhesion is also necessary for cell differentiation [37, 38]. Integrin expression promotes DE differentiation from human pluripotent stem cells [39]. Integrin is a receptor of extracellular matrix expressed in the cells, which enables binding of the cells to the extracellular matrix. Itgα5 and ItgαV are DE-specific *Integrins*. Knockdown of either Itgα5 or ItgαV inhibits DE differentiation [39]. Both Daf1- and Daf1 + DE cells express *Itgα5* and *ItgαV*. We found that the expressions of *Itgα1*, *Itgα3,* and *Itgα8* decreased in Daf1 + DE cells. Itgα1 is an attachment molecule of the DE [40]. *Itgα3* is expressed in the DE and expression decreases in the *Foxa2* null mouse embryo [41]. *Itgα8* null mice have abnormal lung morphogenesis [42]. These Daf1-DE specific integrins could regulate DE differentiation and modulate their behavior. The integrins are known to show distinct ligand binding specificities among the superfamily members. *Itgα1β1*, *Itgα3β1* bind specifically to laminin and *Itgα8β1* binds specifically to fibronectin [43]. The lowered expression of *Itgα1*, *Itgα3* might explain the decreased adhesion of Daf1 + DE cells to Matrigel, which composed mainly of laminin. The decrease in adhesion to matrix might reflect the developmental transition from early to late DE.

We found that Daf1-DE could turn into Daf1 + DE cells. Sox17 expression was higher in Daf1 + DE than in Daf1-DE cells. Furthermore, expression of a primitive streak marker, *Brachyury* [44], was higher in Daf1-DE cells (SO unpublished). Therefore, E-cadherin+/Cxcr4+ DE could be a mixed population of both primitive streak and DE cells.

## Conclusions

Our data indicate that DE can be divided into two stages: early and late DEs. Early DE consists of E-cadherin + Cxcr4 + Daf1-Foxa2 + Sox17^low^ cells that show higher proliferative activity and higher cell-matrix adhesive capacity. Later on, these DE cells differentiate into E-cadherin + Cxcr4 + Daf1+ Foxa2 + Sox17^high^ late DE cells that show a decreased proliferation and low cell-matrix adhesion capacity.

Our findings would contribute to the understandings of the differentiation of the primitive streak and DE during gastrulation.

## Methods

### Cell lines

ESC cell lines (R1, SK7 *Pdx1*/GFP) [25] or a mouse Nanog iPS cell line (20D-17) [45], were maintained on a feeder layer of mouse embryonic fibroblasts (MEFs) in Dulbecco’s Modified Eagle Medium (DMEM) (Invitrogen) supplemented with human recombinant LIF (1:1000, Wako), 10 % fetal bovine serum (FBS, Hyclone), 2 mM l-glutamine (l-Gln, Nacalai Tesque), 100 mM non-essential amino acids (NEAA, Invitrogen), 50 U/mL penicillin, 50 mg/ml streptomycin (PS, Nacalai Tesque), and 100 μM 2-mercaptoethanol (2-ME, Sigma-Aldrich) in 5 % CO_2_.

### Differentiation of ESCs

DE differentiation: ESCs or iPSCs (10^4^ cells/ml) were seeded onto mitomycin C (Sigma)-treated M15 feeders, and cultured in the presence of 10 ng/ml Activin (R&D systems) in DMEM containing 10 % FBS, 2 mM l-Gln, 100 mM NEAA, PS, and 100 μM 2-ME. Pancreatic differentiation: Sorted DE cells were seeded onto MEF feeders and cultured with DE differentiation medium supplemented with 5 ng/ml FGF2 (Peprotech). Intestinal differentiation: Sorted DE cells were seeded onto MEF feeders and cultured with DMEM (2000 mg/ml glucose) and 5 μM bromoindirubin-3′-oxime (BIO) (Wako), 10 μM *N*-[(3, 5-diflurophenyl) acetyl]- l-alanyl-2-phenylglycine-1, 1-dimethylethyl ester (DAPT) (Peptide), 10 % Knockout Serum Replacement (KSR)(In vitrogen), 2 mM l-Gln, 100 mM NEAA, PS, and 100 μM 2-ME.

### Microarray analysis

Our previously described results were used in the present study [22, 23].

### Antibodies

For immunocytochemical analysis, goat anti-Sox17 antibody (1:100, R&D systems), rabbit anti-Hnf3b/Foxa2 (1:200, Millipore), mouse anti-Cdx2 (1:500, BioGenex) and rabbit anti-GFP (1:1000, MBL) were used. For flow cytometric analysis, rat anti-E-cadherin (1:500, TaKaRa), biotin anti-Cxcr4 (1:500, BD Biosciences), PE anti-CD55/Daf1 (1:100, BD Biosciences), PE/Cy7 Streptavidin (1:500, Biolegend) antibodies were used. E-cadherin antibody was labeled by Allophycocyanin Labelling Kit-SH2 (DOJINDO). For Western blot analysis, mouse anti-α-tubulin (1:2000, 12G10, Developmental Studies Hybridoma Bank), mouse anti-phospho-Histone H3 (Ser10) antibody (1:500, Millipore), rabbit anti-Sox17 antibody (1:100, Sigma-Aldrich) and mouse anti-PCNA (1:500, Oncogene, NA03-200UG) were used.

### Immunocytochemical analysis

Cells were fixed with 4 % paraformaldehyde (PFA) (Nacalai Tesque) for 5 min. After fixation, cells were permeabilized with 0.1 % TritonX (Nacalai Tesque) for 10 min. Then, cells were blocked with Blocking One (Nacalai Tesque) and stained with antibodies.

### Flow cytometry analysis

Cells were dissociated with Cell Dissociation Buffer (Invitrogen) and stained with the appropriate antibodies. The stained cells were recovered using FACS Aria II (BD Biosciences). Data were recorded using the BD FACS Diva Software program (BD Biosciences) and analyzed using the FlowJo program (Tree Star).

### Western blot analysis

Cells were homogenized in SDS sample buffer (62.5 mM Tris–HCl, 10 % glycerol, 2 % SDS, pH 6.8). After centrifugation, the supernatants were collected and used as total protein extracts. Total proteins were subjected to 8 % SDS-PAGE and transferred to polyvinylidene difluoride membranes (Immobilon-P Transfer Membrane, Millipore). The membranes were incubated with antibodies listed above. Horseradish peroxidase (HRP) conjugated anti-rabbit IgG (1:20000, CST) was used as the secondary antibody. Chemiluminescence signals were detected with ECL Plus Western Blotting Detection Reagents (GE Healthcare, Japan).

### RT-PCR analysis

RNA was extracted from the cells using the RNeasy Micro-Kit (QIAGEN) and then 1 μg of RNA was reverse transcribed using ReverTra Ace (TOYOBO), ribonuclease inhibitor, recombinant (TOYOBO), and Oligo dT primers (TOYOBO). Primer sequences are shown in Additional file 3.

### Cell cycle analysis

Cells were dissociated with Cell Dissociation Buffer (Gibco). Dissociated cells were washed with PBS and treated with Vybrant DyeCycle Violet Stain (Life Technologies) for 30 min at 37 °C. Cells were analyzed by FACS Canto (BD Biosciences).

### Cell- matrix adhesion analysis

The sorted cells were plated onto matrigel-precoated dishes with serum free medium for 90 min. The attached cells were fixed with 4 % PFA for 5 min, then stained with DAPI (1:2000, Roche). Cell counts were performed as previously described [46].

### Apoptosis assay

For the apoptosis assay, caspase-3/7 activity was measured using CellEvent™ Caspase-3/7 Green Detection Reagent (Invitrogen Life Technologies Co., Carlsbad, CA, USA) according to the manufacturer’s protocol.

## Abbreviations

2-ME, β-mercaptoethanol; APS, anterior primitive streak; Cxcr4, chemokine (C-X-C motif) receptor 4; Daf1/CD55, decay accelerating factor 1; Daf1-DE, Daf1-negative DE cells; DAPI, 2-(4-amidinophenyl)-1H -indole-6-carboxamidine; DE, definitive endoderm; DMEM, Dulbecco’s modified eagle medium; ESC, embryonic stem cells; FBS, fetal bovine serum; *FoxA2*, *Forkhead box A2*; GFP, green fluorescent protein; HRP, horseradish peroxidase; iPSC, induced pluripotent stem cell, Daf1 + DE, Daf1-positive DE cells; KSR, knockout serum replacement; l-Gln, l-glutamine; MEF, mouse embryonic fibroblasts; NEAA, non-essential amino acids; PCNA, proliferating cell nuclear antigen; PFA, paraformaldehyde; PH3, phosphorylated histone H3; PS, penicillin & streptomycin; RT-PCR, reverse transcription-polymerase chain reaction; *Sox17*, *sex-determining region Y-box 17*

## Additional files

Additional file 1: Daf1-positive cells are negative for Nanog expression. Mouse Nanog-iPS cells, in which GFP expression is driven by *Nanog* promoter [45], are differentiated into DE. Cxcr4+/E-cadherin + cells were sorted and analyzed for Daf1 and Nanog-GFP expression. Daf1-positive cells are negative for Nanog expression. (TIF 312 kb) Additional file 2: Flow cytometric analyses of the cell cycle. Histograms of flow cytometric analyses of Daf1-DE and Daf1 + DE (n = 5) are shown. Cell cycle was analyzed by measuring DNA quantities using DyeCycle. (TIF 314 kb) Additional file 3: Primer sequences used for RT-PCR analysis. Primer sequences used for detection of gene expression in Fig. 1, 2. (DOCX 15 kb)
