# Supplementary figures and images for: Late stage definitive endodermal differentiation can be defined by Daf1 expression
Source: BMC Dev Biol. 2016 May 31;16:19. doi: 10.1186/s12861-016-0120-2 (PMC4888667; doi:10.1186/s12861-016-0120-2)

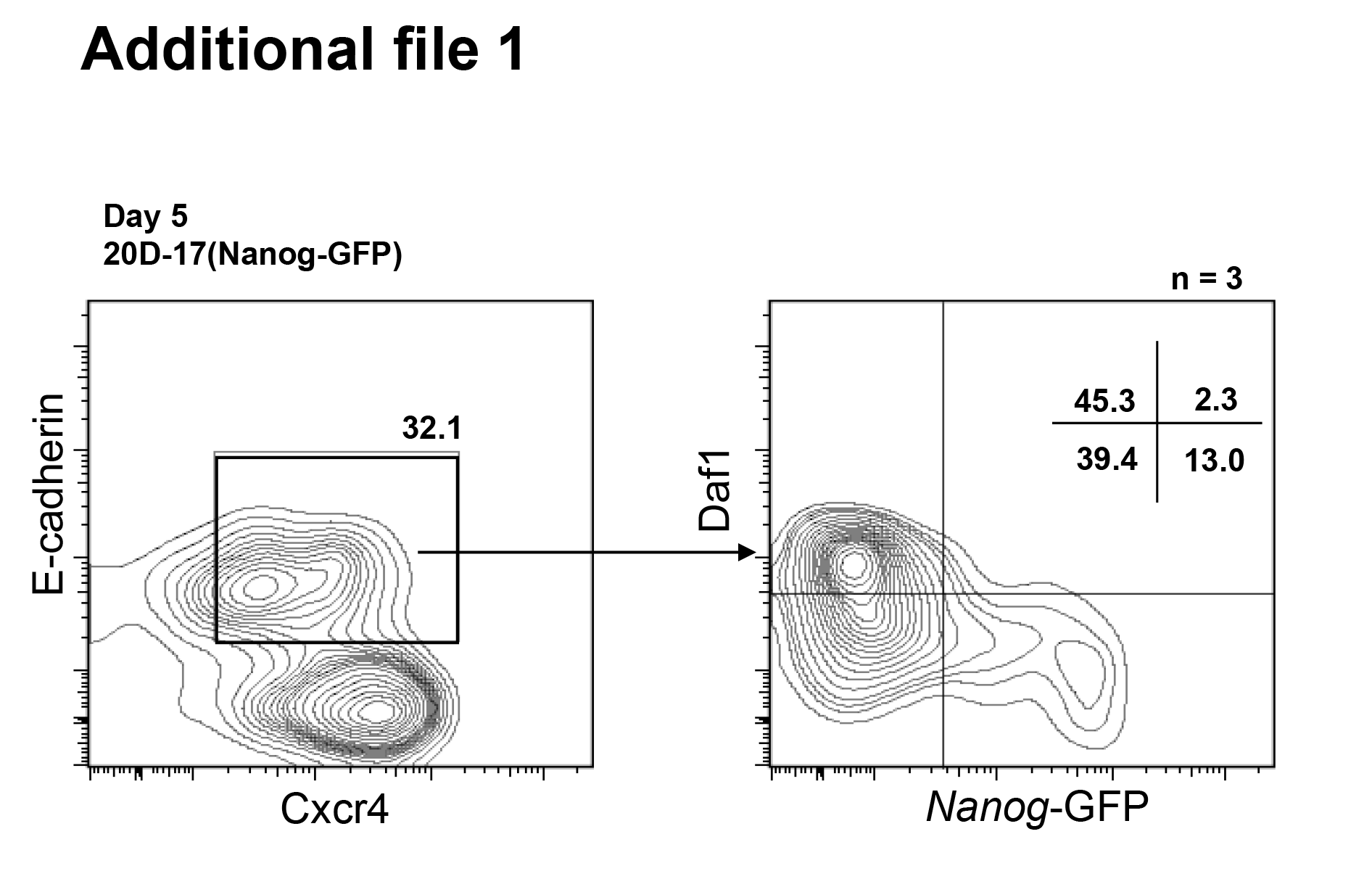

Supplement: Additional file 1: — Daf1-positive cells are negative for Nanog expression. Mouse Nanog-iPS cells, in which GFP expression is driven by Nanog promoter [45], are differentiated into DE. Cxcr4+/E-cadherin + cells were sorted and analyzed for Daf1 and Nanog-GFP expression. Daf1-positive cells are negative for Nanog expression. (TIF 312 kb) [file 12861_2016_120_MOESM1_ESM.tif]

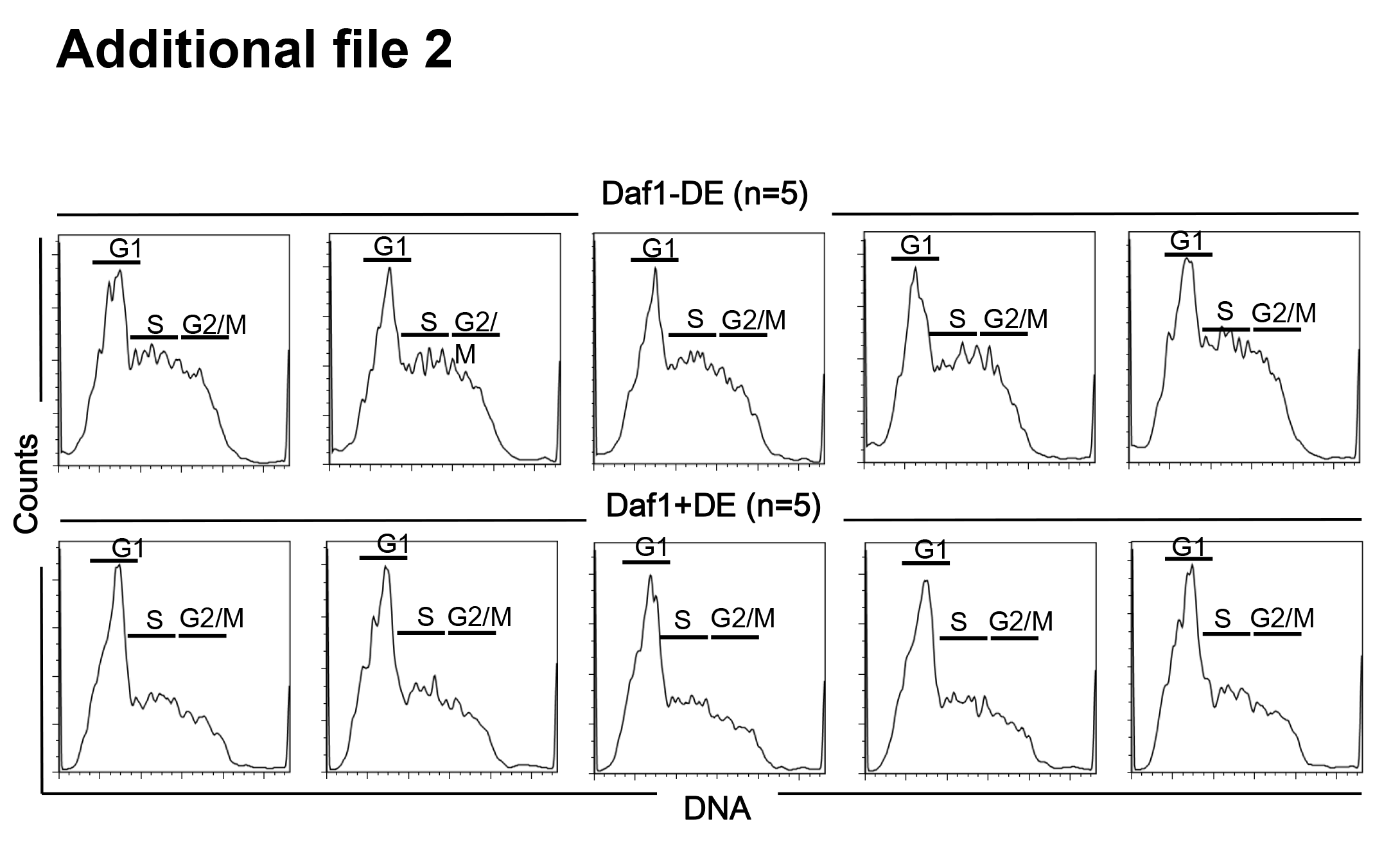

Supplement: Additional file 2: — Flow cytometric analyses of the cell cycle. Histograms of flow cytometric analyses of Daf1-DE and Daf1 + DE (n = 5) are shown. Cell cycle was analyzed by measuring DNA quantities using DyeCycle. (TIF 314 kb) [file 12861_2016_120_MOESM2_ESM.tif]
