# Supplementary material for: Late stage definitive endodermal differentiation can be defined by Daf1 expression
Source: BMC Dev Biol. 2016 May 31;16:19. doi: 10.1186/s12861-016-0120-2 (PMC4888667; doi:10.1186/s12861-016-0120-2)
Supplement: Additional file 3: — Primer sequences used for RT-PCR analysis. Primer sequences used for detection of gene expression in Fig. 1, 2. (DOCX 15 kb) [file 12861_2016_120_MOESM3_ESM.docx]

**Additional file 3 Primer sequences used for RT-PCR analysis**

| **Gene** | **Forward Primer** | **Reverse Primer** |
| --- | --- | --- |
| β-actin | GTGATGGTGGGAATGGGTCA | TTTGATGTCACGCACGATTTCC |
| Daf1 | CCAGAGCCACCAAAAATCAA | TCCTACATCAGACTTGCTCACAG |
| Itgβ1 | TTGGGATGATGTCGGGAC | AATGTTTCAGTGCAGAGC |
| Itgβ5 | GGTTTCGGGTCTTTTGTTGA | GCTTCCTCACTTCCTCGTTG |
| Itgβ8 | ACAGACCAGACATCACATCTTGCTC | AATGAAACTGCTTTCCTTGAACTGC |
| Itgα1 | GGGCAGCCTCGGTACAATCA | AGCCCCGACGAGAAGCAGAT |
| Itgα2 | CCATGATGGGTCGAAGCTGA | CTTCGTCGGCCACATTGAAA |
| Itgα3 | TGTGTACCTGTGTCCCCTCA | CTTCTAGCCCAGACCACAGC |
| Itgα4 | GAGAATTGAAGGACAGCAAATCAGC | ATGCACCAATGGCTACATCAACATA |
| Itgα5 | AGCGACTGGAATCCTCAAGA | TGCTGAGTCCTGTCACCTTG |
| Itgα6 | AGCCCCAGGGACTTACAACT | CTCTTGGAGCACCAGACACA |
| Itgα7 | CTCTCCCAGCCTCTCTACGGTACTC | CAGAGGTGCTGAGGATGAGGTAAAA |
| Itgα8 | CGAGGTGCAGTTAGATTTCCTGAAG | TGTAGTTCAGGCTGATGTTGATTGG |
| Itgα9 | GAAAGGAATTGCCAATCTGAGGACT | CCTGGAGACATTAAAGGACACGTTG |
| Itgα10 | CTCTGCTCCCCCTTTAATCTGGATG | GAGCAACGATAAACATCCCCTCTCC |
| Itgα11 | GGAGTTTCCAGAGGAGCTGAAGAAC | AGAATGACCTTGCCAGTATGGTTGA |
| ItgαV | GGGTGATCATCTTGGCAGTT | GAACTTGGAGCGGACAGAAG |

Primer sequences used for detection of gene expression in Fig. 1 & Fig. 2.
